# Pseudo-Qutrit Formed by Two Interacting Identical Spins (s = 1/2) in a Variable External Magnetic Field

**DOI:** 10.3390/e25050716

**Published:** 2023-04-26

**Authors:** Yury Belousov, Igor Chernousov, Vladimir Man’ko

**Affiliations:** 1 Terra Quantum AG, St. Gallerstrasse 16A, 9400 Rorschach, Switzerland; 2 Moscow Institute of Physics and Technology, Institutskiy Per. 9, Moscow Distr., Dolgoprudny 141701, Russia; 3 Lebedev Physical Institute, Leninskii Prospect 53, Moscow 119991, Russia

**Keywords:** spin 1/2, magnetic dipole–dipole interaction, pseudo-qutrit, transition probability, entangled states

## Abstract

An analytical solution is obtained for the problem of two interacting, identical but separated spin 1/2 particles in a time-dependent external magnetic field, in a general case. The solution involves isolating the pseudo-qutrit subsystem from a two-qubit system. It is shown that the quantum dynamics of a pseudo-qutrit system with a magnetic dipole–dipole interaction can be described clearly and accurately in an adiabatic representation, using a time-dependent basis set. The transition probabilities between the energy levels for an adiabatically varying magnetic field, which follows the Landau–Majorana–Stuckelberg–Zener (LMSZ) model within a short time interval, are illustrated in the appropriate graphs. It is shown that for close energy levels and entangled states, the transition probabilities are not small and strongly depend on the time. These results provide insight into the degree of entanglement of two spins (qubits) over time. Furthermore, the results are applicable to more complex systems with a time-dependent Hamiltonian.

## 1. Introduction

Spin systems are of increasing interest due to their potential applications in quantum information and related technologies [1,2]. In the field of quantum computing, spin Hamiltonian models provide a theoretical foundation for manipulating two-electron-based qubits, such as those in a double quantum dot [3,4,5,6,7] or a double quantum well [8,9].

A two-level spin system (s = 1/2) serves as a classical prototype for a qubit, the basic unit of quantum information. Spin systems influenced by hyperfine interactions have been widely studied due to their relevance to phenomena such as NMR and ESR, as well as related spectroscopic techniques. The potential for using spin systems in quantum information and computation has opened up a broad range of previously unsolved dynamical problems that are not typically considered in standard NMR and ESR applications. Adiabatic gates, based on the well-known adiabatic approximation, play an important role in quantum information processing [10,11,12,13].

The problem of two interacting identical spins is of interest in quantum information technology because the problems being studied differ from those traditionally addressed in NMR research. In traditional NMR, a high external magnetic field limit is used, with a nuclear magnetic dipole–dipole interaction treated as a small perturbation. This perturbation can yield interesting results for research on paramagnetic radicals or other more complex magnetic systems beyond two interacting identical nuclear spins. However, interesting results for the preparation of entangled states of two nuclear spins can also be obtained using weak external magnetic fields comparable to nuclear magnetic fields.

The problem of two interacting neighboring nuclear spins in a crystal lattice is of interest. The most frequently used nuclei isotopes with nonzero spins I=1/2 are ${}^{13}$C with a magnetic moment $\mu_{C}=+0.70238\mu_{n},\;1.108\%$, ${}^{15}$N with $\mu_{N}=-0.283049\mu_{n},\;0.365\%$. Naturally, we must not forget hydrogen with “the main” nuclear isotope ${}^{1}$H with $\mu_{p}=+2.79867\mu_{n}$, where the nuclear magneton $\mu_{n}=5.05095\cdot 10^{-24}$ Erg/G. Isotopes ${}^{13}$C and ${}^{15}$N are interesting because they are the main impurities in diamond crystals, and are popular in many research studies on quantum technologies. Silicon, another popular crystal for quantum technologies, has the same diamond lattice structure. The isotope ${}^{29}$Si with a nonzero nuclear spin I=1/2 has a magnetic moment of $\mu_{Si}=-0.55\mu_{n}$ and a prevalence in nature of 4.67%.

The problem could also be of interest for “frozen” diatomic molecules with identical nuclei, such as the hydrogen molecule H${}_{2}$, or more complicated molecules with a pair of the two nearest protons or other identical isotopes. Such molecules must be frozen to exclude averaging of the direct nuclear magnetic dipole–dipole interaction over states with certain angular momenta of the molecule. In this case, there is no connection between the spin and rotational states of the nuclei due to the Pauli principle.

Recently, the problem of two interacting identical nuclear spins situated in a crystal lattice as nearest neighbors or in some other “frozen” state has been studied [14,15]. In [15], it was shown that the formation of a pseudo-qutrit state is possible for identical spin 1/2 particles. Furthermore, the problem was solved for both standard quantum and probability representations of quantum states [16,17], where spin states were described by standard classical probability distributions [18,19,20] in the stationary case. However, for processing quantum technology elements, it is interesting to solve the problem of nonstationary situations, such as when an external magnetic field varies over time. The problem of interacting inequivalent spin 1/2 particles in a variable magnetic field was examined in [21] for two partial cases where an adiabatic representation was introduced.

Here, we address the behavior of two separated but identical spin 1/2 particles with dipole–dipole interactions in a variable external magnetic field.

The paper is organized as follows. Section 1 analyzes the Hamiltonian and states of the system in a stationary case, with a separation of pseudo-qutrit states. Section 2 describes the states in the zero magnetic field in terms of the rotation operator. Section 3 considers an approach for the approximate diagonalization of the Hamiltonian matrix, and Section 4 shows approximate energy levels. Adiabatic representation is introduced in Section 5, and transitions between energy levels and states of a pseudo-qutrit are presented in Section 6. The obtained results and their applications are discussed in Section 7. The conclusions section presents the following developments.

## 2. Hamiltonian and Separation into Two Subspaces

We use the Hamiltonian in frequency units, where ℏ=1. The Hamiltonian for two separated equivalent nuclear spin 1/2 particles can be expressed as follows:

(1) $$\hat{H}=-\omega \left({\hat{s}}_{1z}+{\hat{s}}_{2z}\right)+A_{ik}{\hat{s}}_{1i}{\hat{s}}_{2k},$$

where the external magnetic field B∥z and $\omega =g\mu_{0}B$, *g* is a nuclear *g*-factor, $\mu_{0}$ is the appropriate magneton. The hyperfine interaction tensor can be written as

(2) $$A_{ik}=-\frac{{\left(g\mu_{0}\right)}^{2}}{R^{3}}\left(3n_{i}n_{k}-\delta_{ik}\right).$$

Here, *R* denotes the modulus of the radius vector between the two spins and $n_{i}$ represents the components of the unit vector n=R/R.

The Hamiltonian (1) can be rewritten as follows:

(3) $$\hat{H}=-\omega \left({\hat{s}}_{1z}+{\hat{s}}_{2z}\right)-3\Omega \left(n{\hat{s}}_{1}\right)\left(n{\hat{s}}_{2}\right)+\Omega \left({\hat{s}}_{1}{\hat{s}}_{2}\right),$$

where $\Omega ={\left(g\mu_{0}\right)}^{2}/R^{3}$. We can see that this expression corresponds to the hyperfine interaction Hamiltonian with an axial symmetry examined in [21].

If we choose a non-conventional enumeration of a standard base of state vectors

(4) $$|\chi_{1}\rangle =|+\rangle |+\rangle ,\;|\chi_{2}\rangle =|-\rangle |-\rangle ,\;|\chi_{3}\rangle =|-\rangle |+\rangle ,\;|\chi_{4}\rangle =|+\rangle |-\rangle ,$$

the Hamiltonian Matrix (3) is written as follows

(5) $$H=\left(\begin{array}{cccc} \frac{\Omega }{4}\left(1-3\cos^{2}\theta \right)-\omega & -\frac{3\Omega }{4}\sin^{2}\theta & -\frac{3\Omega }{8}\sin2\theta & -\frac{3}{8}\Omega \sin2\theta \\ -\frac{3\Omega }{4}\sin^{2}\theta & \frac{\Omega }{4}\left(1-3\cos^{2}\theta \right)+\omega & \frac{3\Omega }{8}\sin2\theta & \frac{3\Omega }{8}\sin2\theta \\ -\frac{3\Omega }{8}\sin2\theta & \frac{3\Omega }{8}\sin2\theta & -\frac{\Omega }{4}\left(1-3\cos^{2}\theta \right) & \frac{\Omega }{4}\left(2-3\sin^{2}\theta \right)\\ -\frac{3\Omega }{8}\sin2\theta & \frac{3\Omega }{8}\sin2\theta & \frac{\Omega }{4}\left(2-3\sin^{2}\theta \right) & -\frac{\Omega }{4}\left(1-3\cos^{2}\theta \right) \end{array}\right).$$

First, we need to write the energy levels for the zero magnetic field:

(6) $$\varepsilon_{1,2}=-\frac{\Omega }{2},\;\varepsilon_{3}=\Omega \;\mathrm{and}\;\varepsilon_{4}=0.$$

As well known, the problem has exact solutions in two partial cases when θ=0 and π/2. Indeed, for the first case when θ=0, the Hamiltonian matrix on the basis of (4) is equal to

(7) $$H\left(\theta =0\right)=\left(\begin{array}{cccc} -\omega -\Omega /2 & 0 & 0 & 0\\ 0 & \omega -\Omega /2 & 0 & 0\\ 0 & 0 & \Omega /2 & \Omega /2\\ 0 & 0 & \Omega /2 & \Omega /2 \end{array}\right).$$

Eigenvalues can easily be found

(8) $$\varepsilon_{1,2}=-\frac{\Omega }{2}\mp \omega ,\;\varepsilon_{3}=\Omega ,\;\varepsilon_{4}=0,$$

and eigenstates coincide with the total spin S=1 eigenstates (see Equation (14) below).

For the second case, θ=π/2 we obtain the following Hamiltonian matrix

(9) $$H\left(\theta =\pi /2\right)=\left(\begin{array}{cccc} -\omega +\Omega /4 & -3\Omega /4 & 0 & 0\\ -3\Omega /4 & \omega +\Omega /4 & 0 & 0\\ 0 & 0 & -\Omega /4 & -\Omega /4\\ 0 & 0 & -\Omega /4 & -\Omega /4 \end{array}\right)$$

The eigenvalues of Matrix (9) are equal to

(10) $$\varepsilon_{1,2}=\frac{\Omega }{4}\mp \sqrt{{\left(\frac{3\Omega }{4}\right)}^{2}+\omega^{2}},\;\varepsilon_{3}=-\frac{\Omega }{2},\;\varepsilon_{4}=0.$$

Eigenstates are determined by the following expressions:

$$\begin{array}{c} |\varepsilon_{1}\rangle =\frac{1}{\sqrt{2}}\left(\sqrt{1+\frac{x}{\sqrt{9+x^{2}}}}|+\rangle |+\rangle +\sqrt{1-\frac{x}{\sqrt{9+x^{2}}}}|-\rangle |-\rangle \right),\\ |\varepsilon_{2}\rangle =\frac{1}{\sqrt{2}}\left(\sqrt{1+\frac{x}{\sqrt{9+x^{2}}}}|-\rangle |-\rangle -\sqrt{1-\frac{x}{\sqrt{9+x^{2}}}}|+\rangle |+\rangle \right),\\ |\varepsilon_{3}\rangle =\frac{1}{\sqrt{2}}(|+\rangle |-\rangle +|-\rangle |+\rangle ). \end{array}$$

We can achieve progress in the problem’s solution if we make an approximate diagonalization of Hamiltonian Matrix (5) by a unitary transformation. This procedure was successfully carried out for a similar problem in [22] and was further developed in [23]. This approach was also applied to nonstationary problems in [21], where a new adiabatic representation was introduced.

As was shown in [15], the square of the total spin commutes with the Hamiltonian (5); thus, first, we need to choose a base set with a determined total spin $\hat{S}={\hat{S}}_{1}+{\hat{S}}_{2}$, which is achieved with the following transformation matrix

(11) $$\hat{T}=\left(\begin{array}{cccc} 1 & 0 & 0 & 0\\ 0 & 1 & 0 & 0\\ 0 & 0 & 1/\sqrt{2} & 1/\sqrt{2}\\ 0 & 0 & -1/\sqrt{2} & 1/\sqrt{2} \end{array}\right).$$

The transformed Hamiltonian matrix takes the following form

(12) $$\begin{array}{cc} \; & \hat{\tilde{H}}=\hat{T}\hat{H}{\hat{T}}^{†}=\\ \; & \frac{\Omega }{4}\left(\begin{array}{cccc} 1-3\cos^{2}\theta -x & -3\sin^{2}\theta & -\frac{3}{\sqrt{2}}\sin2\theta & 0\\ -3\sin^{2}\theta & 1-3\cos^{2}\theta +x & \frac{3}{\sqrt{2}}\sin2\theta & 0\\ -\frac{3}{\sqrt{2}}\sin2\theta & \frac{3}{\sqrt{2}}\sin2\theta & -2(1-3\cos^{2}\theta ) & 0\\ 0 & 0 & 0 & 0 \end{array}\right). \end{array}$$

Here, a dimensionless variable *x* is introduced as follows

(13) $$x=\frac{4\omega }{\Omega }$$

After this transformation, the problem can be reduced to two separate subsystems. One of them can be described as a system with a well-determined total spin of S=0. The other subsystem is a three-level system for which only the square of the total spin is well-defined. Hence, it can be regarded as one of the possible representations of qutrit states. As a result, the system of two interacting spin 1/2 particles can be decomposed into a scalar subsystem and a subsystem with an effective spin of S=1, representing a qutrit. It was previously shown in [14] that this situation exists for two non-identical spins of 1/2 with scalar hyperfine interaction. For two identical spins, this holds true for any magnetic dipole–dipole interaction. To proceed, it is useful to express the base states in which this separation can be realized. The qutrit states are

(14) $$|q_{1}\rangle =|+\rangle |+\rangle ,\;|q_{2}\rangle =|-\rangle |-\rangle ,\;|q_{3}\rangle =\frac{1}{\sqrt{2}}(|-\rangle |+\rangle +|+\rangle |-\rangle ),$$

and singlet is determined by

(15) $$|0,0\rangle =\frac{1}{\sqrt{2}}(|+\rangle |-\rangle -|-\rangle |+\rangle ).$$

As shown in [15], a density matrix $\rho^{\left(1,0\right)}$ of the two identical spins of 1/2 in bases (14) and (15) can be represented as a direct sum of density matrices in two independent subspaces

(16) $$\rho^{\left(1,0\right)}=\left(\rho^{\left(1\right)}⊕\rho^{\left(0\right)}\right),$$

where $\rho^{\left(1\right)}$ and $\rho^{\left(0\right)}$ are the density matrices of the qutrit and scalar subsystems, respectively. These matrices satisfy the obvious requirement that $\mathrm{Tr}\rho^{\left(1\right)}=1$ and $\mathrm{Tr}\rho^{\left(0\right)}=1$. The explicit form of the density Matrix (16) can be represented as a 4×4 matrix by

(17) $$\rho^{\left(1,0\right)}=\left(\begin{array}{cccc} r_{1} & r_{4} & r_{5} & 0\\ {r_{4}}^{*} & r_{2} & r_{6} & 0\\ {r_{5}}^{*} & {r_{6}}^{*} & r_{3} & 0\\ 0 & 0 & 0 & \alpha \end{array}\right).$$

Here, α is a constant that should be determined from the initial conditions.

In these subspaces, the scalar subsystem does not evolve in time, i.e., $\rho^{\left(0\right)}\left(t\right)=\rho^{\left(0\right)}\left(0\right)$, and the time dependence of the qutrit density matrix is determined from the Liouville equation

(18) $$\frac{\partial \rho^{\left(1\right)}}{\partial t}+\frac{i}{\hbar }\left[{\hat{H}}^{\left(1\right)},\rho^{\left(1\right)}\right]=0$$

where the Hamiltonian ${\hat{H}}^{\left(1\right)}$ is determined by the 3×3 matrix in the base state (14)

(19) $${\hat{H}}^{\left(1\right)}=\frac{\Omega }{4}\left(\begin{array}{ccc} 1-3\cos^{2}\theta -x & -3\sin^{2}\theta & -\frac{3}{\sqrt{2}}\sin2\theta \\ -3\sin^{2}\theta & 1-3\cos^{2}\theta +x & \frac{3}{\sqrt{2}}\sin2\theta \\ -\frac{3}{\sqrt{2}}\sin2\theta & \frac{3}{\sqrt{2}}\sin2\theta & -2(1-3\cos^{2}\theta ) \end{array}\right).$$

## 3. The Rotation Operator of Identical Spin 1/2 Particles

It is easy to see that the energy levels of the pseudo-qutrit system depend on the relative orientations of the two dipoles with respect to the external magnetic field in the general case, but they cannot depend on the orientation when the external field is zero. Indeed, in the absence of an external magnetic field, we have one doubly degenerate level and one non-degenerate. If we follow the numbering of state (8), we obtain $\varepsilon_{1}=\varepsilon_{2}=-2$ and, accordingly, $\varepsilon_{3}=4$. If we follow the numbering of state (10), we have further correspondence, i.e., $\varepsilon_{1}=\varepsilon_{3}=-2$ and, respectively, $\varepsilon_{2}=4$. In other words, after changing the direction of the quantization axis, the second and third states swap $|\varepsilon_{2}\rangle ⇆|\varepsilon_{3}\rangle$. At the same time, the eigenstates are equal

(20) $$|\varepsilon_{1,2}\rangle =\pm \frac{1}{\sqrt{2}}\left(|+\rangle |+\rangle \pm |-\rangle |-\rangle \right).$$

As one can see, in the first case, only the state with zero projection of the total spin is entangled, but in the second case, all states are entangled.

On the other hand, the second case follows from the first as a result of the rotation of the quantization axis *z* by an angle π/2 relative to the axis *y*. Thus, the eigenstates *with arbitrary mutual orientation* of the magnetic field and the axis n can be obtained as a result of the initial state by a corresponding rotation transformation relative to the axis *z*, when B∥n. However, the problem is not so simple because we need to know the quantization axis direction in an arbitrary case. To do this, we need to solve the problem. Indeed, in an arbitrary case, the quantization axis does not coincide with the magnetic field direction. However, only in the case x≫1 can one believe that the quantization axis practically coincides with the magnetic field direction.

For these reasons, we consider the rotation operator for two equivalent Fermi particles with spins (s=1/2) with respect to the *y*-axis by angle 2β. The rotation operator is equal to

(21) $${\hat{R}}_{y}^{\left(1\right)}\left(\beta \right)=e^{i\beta (\sigma_{1y}+\sigma_{2y})}.$$

We decompose the exponent into a Taylor series, noting that

(22) $${\left(\sigma_{1y}+\sigma_{2y}\right)}^{2}=2\left(1+\sigma_{1y}\sigma_{2y}\right),\;{\left(\sigma_{1y}+\sigma_{2y}\right)}^{3}=4\left(\sigma_{1y}+\sigma_{2y}\right).$$

Thus, the rotation operator (21) is transformed as follows

(23) $${\hat{R}}_{y}^{\left(1\right)}\left(\beta \right)=\frac{1}{2}\left(1+\cos\beta \right)+\frac{1}{2}\left(1-\cos\beta \right)\left(\sigma_{1y}\sigma_{2y}\right)+\frac{i}{2}\sin\beta \left(\sigma_{1y}+\sigma_{2y}\right).$$

Equation (23) is valid for the rotation operator, with respect to any other axis with the corresponding replacement of the projections of the spin operators.

By acting with the rotation operator (23) on state (14), we have the following expressions:

(24) $$\begin{array}{cc} {\hat{R}}_{y}^{\left(1\right)}\left(\beta \right)|+\rangle |+\rangle = & \frac{1}{2}\left(1+\cos\beta \right)|+\rangle |+\rangle +\frac{1}{2}\left(1-\cos\beta \right)|-\rangle |-\rangle -\frac{1}{2}\sin\beta (|+\rangle |-\rangle +|-\rangle |+\rangle ),\\ {\hat{R}}_{y}^{\left(1\right)}\left(\beta \right)|-\rangle |-\rangle = & \frac{1}{2}\left(1+\cos\beta \right)|-\rangle |-\rangle +\frac{1}{2}\left(1-\cos\beta \right)|+\rangle |+\rangle +\frac{1}{2}\sin\beta (|+\rangle |-\rangle +|-\rangle |+\rangle ),\\ {\hat{R}}_{y}^{\left(1\right)}\left(\beta \right)|\varepsilon_{3}\rangle = & \cos\beta |\varepsilon_{3}\rangle +\frac{1}{\sqrt{2}}\sin\beta (|+\rangle |+\rangle -|-\rangle |-\rangle ). \end{array}$$

Note that in a zero magnetic field, the energy level −Ω/2 is doubly degenerated, so any linear superposition of states $|\varepsilon_{1}\rangle$ and $|\varepsilon_{2}\rangle$ will also be an eigenstate. For state (14), a ‘natural’ quantization axis is selected that coincides with the direction of the axis connecting the two interacting spins. However, due to the symmetry of the interaction, the energy can only depend on the projection module. The state of $|\varepsilon_{3}\rangle$ is uniquely determined because the energy level of $\varepsilon_{3}$ is non-degenerate. Thus, we write linear combinations as follows

(25) $$\begin{array}{cc} |1\rangle & =a|+\rangle |+\rangle +b|-\rangle |-\rangle =\frac{1}{\sqrt{2}}(|+\rangle |+\rangle +|-\rangle |-\rangle ),\\ |2\rangle & =-b|+\rangle |+\rangle +a|-\rangle |-\rangle =\frac{1}{\sqrt{2}}(|-\rangle |-\rangle -|+\rangle |+\rangle ). \end{array}$$

By applying the rotation operator (23) on state (25), we have

(26) $${\hat{R}}_{y}^{\left(1\right)}\left(\beta \right)|1\rangle |=|1\rangle ),\;{\hat{R}}_{y}^{\left(1\right)}\left(\beta \right)|2\rangle =\cos\beta |2\rangle +\frac{1}{\sqrt{2}}\sin\beta (|+\rangle |-\rangle +|-\rangle |+\rangle ).$$

As one can see, state |1〉 is the proper state of the rotation operator, but state |2〉 becomes a superposition with state $|\varepsilon_{3}\rangle$ at the angle of rotation 2β=π/2 and states |2〉 and $|\varepsilon_{3}\rangle$ pass each other. It corresponds to Solution (10) in the case of a zero external magnetic field.

The symmetry of the system is broken when the external magnetic field is not zero, and the energy depends on the orientation of the two magnetic dipoles relative to the direction of the magnetic field. Therefore, the superposition of state (14) depends on both the angle and the magnitude of the field. The effective angle of rotation is determined by the ratio between the characteristic value of the interaction of two magnetic dipoles, Ω, and the magnitude of the magnetic field, as emphasized above.

## 4. Approximate Diagonalization

To solve the problem, we need to first find the eigenvalues and eigenstates of Matrix (19) (the energy levels and states of the pseudo-qutrit). For the solution, we will apply an approximate diagonalization approach [22,23] and introduce the adiabatic representation [21]. Note, that base states $|q_{1}\rangle$ and $|q_{2}\rangle$ are strongly mixed for θ≠0. To take this mixture into account, let us perform the first transformation $T_{1}$ of Matrix (19) to eliminate non-diagonal elements $H_{12}^{\left(1\right)}$ and $H_{21}^{\left(1\right)}$

(27) $${\hat{T}}_{1}=\left(\begin{array}{ccc} \cos\vartheta_{1} & -\sin\vartheta_{1} & 0\\ \sin\vartheta_{1} & \cos\vartheta_{1} & 0\\ 0 & 0 & 1 \end{array}\right),$$

The transformation parameter $\vartheta_{1}$ is found from the following equation (note that this transformation shows instability for x→0 due to the degeneracy of the energy level in a zero magnetic field):

(28) $$\tan2\vartheta_{1}=\frac{2H_{12}^{\left(1\right)}}{H_{22}^{\left(1\right)}-H_{11}^{\left(1\right)}}=\frac{3\Omega }{4\omega }\sin^{2}\theta =\frac{3}{x}\sin^{2}\theta .$$

For the transformation parameter $\vartheta_{1}$, we have the following relations

$$\cos2\vartheta_{1}=\frac{x}{\sqrt{x^{2}+9y^{2}}},\;\sin2\vartheta_{1}=\frac{3y}{\sqrt{x^{2}+9y^{2}}},$$

where we define $\sin^{2}\theta =y$. In these definitions, the transformed Hamiltonian matrix is written as follows

(29) $$\begin{array}{cc} \; & \hat{\tilde{H}}={\hat{T}}_{1}^{†}\hat{H^{\left(1\right)}}{\hat{T}}_{1}=\\ \; & \frac{\Omega }{4}\left(\begin{array}{ccc} -2+3y-\sqrt{x^{2}+9y^{2}} & 0 & -\frac{3}{\sqrt{2}}\sin2\theta S_{-}\\ 0 & -2+3y+\sqrt{x^{2}{+9y)}^{2}} & \frac{3}{\sqrt{2}}\sin2\theta S_{+}\\ -\frac{3}{\sqrt{2}}\sin2\theta S_{-} & \frac{3}{\sqrt{2}}\sin2\theta S_{+} & 2(2-3y) \end{array}\right). \end{array}$$

where the following designations are introduced

(30) $$S_{\pm }\equiv \cos\vartheta_{1}\pm \sin\vartheta_{1}=\frac{1}{\sqrt{2}}\left(\sqrt{1+\frac{x}{\sqrt{x^{2}+9y^{2}}}}\pm \sqrt{1-\frac{x}{\sqrt{x^{2}+9y^{2}}}}\right).$$

We can see that after the transformation using Matrix (27), the diagonal element ${\tilde{H}}_{11}^{\left(1\right)}$ of Matrix (29) passes to the corresponding energy level in the zero magnetic field and coincides with the exact solution for θ=0 and π/2 in a nonzero magnetic field. Indeed, for these cases, we have:

$${\tilde{H}}_{11}=\left\{\begin{array}{cc} -\Omega /2-x=\varepsilon_{1}, & \mathrm{for}\;\theta =0,\\ \Omega /4-\sqrt{\omega^{2}+{\left(3\Omega /4\right)}^{2}}=\varepsilon_{1}, & \mathrm{for}\;\theta =\pi /2. \end{array}\right.$$

The energy levels of the Hamiltonian (12) strongly depend on the angle θ between the magnetic field and the direction n, resulting in a significantly different picture compared to inequivalent interacting spin 1/2 particles. Nevertheless, this problem presents an opportunity to improve upon the solution for different spins.

For the definition of all three energy levels, we have the third-order algebraic equation, which has a simple form, and is obtained after a relatively cumbersome calculation:

(31) $$\varepsilon^{3}-\left(x^{2}+12\right)\varepsilon +\left(1+3\cos2\theta \right)x^{2}-16=0.$$

We can calculate all eigenvalues in an analytical form; however, these expressions are cumbersome and not convenient for the following solution. Thus, we will continue the process of an approximate diagonalization when we can reduce our problem to the two-level problem. As well-known, a solution for the two-level system can be obtained in a general case [24] (see also [25,26]) and applied to the following solution of the problem.

## 5. Energy Levels

Equation (31) gives the solution for the three energy levels, and we will solve it approximately. As mentioned above, energy level $\varepsilon_{1}$ has correct expressions for two limits, θ=0 and π/2. When we use approximate diagonalization, we compare the difference between two diagonal elements with the appropriate non-diagonal elements of the Hamiltonian matrix. In the transformed Hamiltonian Matrix (29), we can see that only ${\tilde{H}}_{22}-{\tilde{H}}_{33}$ can be equal to zero; the appropriate states will be mixed. Thus, we suppose that in the zero approximation

(32) $$\varepsilon_{1}^{\left(0\right)}\approx {\tilde{H}}_{11}=-2+3y-\sqrt{x^{2}+9y^{2}}.$$

We will find the last two energy levels from the diagonalization of the 2×2 submatrix. Indeed, the obtained results will not satisfy the exact characteristic Equation (31); however, they will correspond with the approximation accepted for energy level $\varepsilon_{1}^{\left(0\right)}$. Thus, we need to solve the problem of searching for eigenvalues and eigenvectors of the matrix

(33) $${\tilde{H}}_{2,3}=\left(\begin{array}{cc} -2+3y+\sqrt{x^{2}{+9y)}^{2}} & \frac{3}{\sqrt{2}}\sin2\theta S_{+}\\ \frac{3}{\sqrt{2}}\sin2\theta S_{+} & 2(2-3y) \end{array}\right).$$

Matrix (33) can be diagonalized with the unitary transformation

(34) $${\hat{T}}_{2}=\left(\begin{array}{cc} \cos\vartheta_{2} & -\sin\vartheta_{2}\\ \sin\vartheta_{2} & \cos\vartheta_{2} \end{array}\right),$$

where the transformation parameter $\vartheta_{2}$ is equal to

(35) $$\tan2\vartheta_{2}=\frac{2\sqrt{2}\sin2\theta S_{+}}{3\left(2-3y\right)-\sqrt{x^{2}+9y^{2}}}.$$

where $S_{+}$ is determined by Equation (31).

We obtain, respectively, cumbersome expressions for the eigenvalues:

(36) $$\begin{array}{cc} \; & \varepsilon_{2,3}^{\left(0\right)}=\frac{1}{2}\left(2-3y+\sqrt{x^{2}+9y^{2}}\right)\pm \\ \; & \frac{1}{2}{\left[9{\left(2-3y\right)}^{2}-6\left(2-3y\right)\sqrt{x^{2}+9y^{2}}+x^{2}+9y^{2}+72y\left(1-y\right)\left(1+\frac{3y}{\sqrt{x^{2}+9y^{2}}}\right)\right]}^{1/2}. \end{array}$$

The obtained relations (36) provide correct expressions for energy levels in two limits, when θ=0 and π/2 and for arbitrary angle θ in the zero external field.

**Figure 1:**
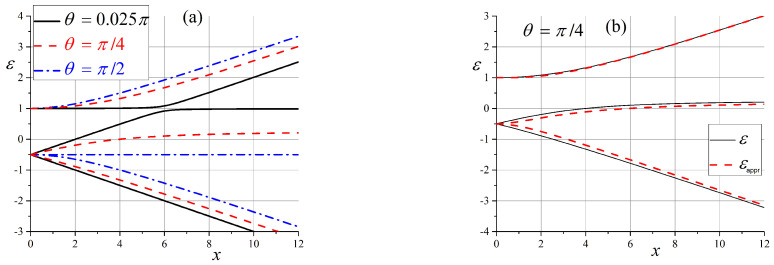
Scheme of energy levels: (**a**) Dependence on the external magnetic field for three different angles θ=0.025π,π/4, and π/2; (**b**) comparison of exact and approximate solutions for the worst case θ≈π/4.

A scheme of energy levels for θ is shown in Figure 1. Even in the worst case, when θ is close to π/4, we have the exact and approximate solutions. This gives us a reason to use approximate analytical solutions to describe the evolution of states in the future.

After this approximate diagonalization, the rest of the diagonal elements of a Hamiltonian matrix after all transformations should be taken into account as small perturbations in frames of perturbation approximations. However, the problem is not so simple when the Hamiltonian depends on time.

## 6. Adiabatic Representation

Now, we will solve the problem in terms of the vector of states |χ(t)〉. The obtained results can be easily applied to a density matrix formulation.

The Schrödinger equation for the state vector in our problem reads

(37) $$i\frac{\partial }{\partial t}|\chi \left(t\right)\rangle =\hat{H}\left(t\right)|\chi \left(t\right)\rangle ,\;|\chi \left(t\right)\rangle =U\left(t\right)|\chi \left(0\right)\rangle ,$$

where the time-dependent Hamiltonian is given by Equation (19), U(t) is the evolution operator, and |χ(0)〉 is the initial state of the system. Thus, the time dependence of the Hamiltonian is contained only in x(t) in the form of a parameter. The evolution operator can be easily found when the Hamiltonian matrix has a diagonal form.

We performed an approximate diagonalization of the Hamiltonian matrix in a stationary case with two unitary matrices

(38) $${\hat{T}}_{t}={\hat{T}}_{1}{\hat{T}}_{2}=\left(\begin{array}{ccc} \cos\vartheta_{1} & -\sin\vartheta_{1}\cos\vartheta_{2} & \sin\vartheta_{1}\sin\vartheta_{2}\\ \sin\vartheta_{1} & \cos\vartheta_{1}\cos\vartheta_{2} & -\cos\vartheta_{1}\sin\vartheta_{2}\\ 0 & \sin\vartheta_{2} & \cos\vartheta_{2} \end{array}\right).$$

As shown, diagonal elements of the transformed matrix ${\hat{T}}_{t}^{†}{\hat{H}}^{\left(1\right)}{\hat{T}}_{t}$ are equal to energy levels, with good accuracy. However, the non-diagonal elements of the transformed Hamiltonian matrix remain, which, if necessary, can later be taken into account as small perturbations:

(39) $${\hat{V}}_{0}=-\frac{3\Omega }{4\sqrt{2}}\sin2\theta \left(\begin{array}{ccc} 0 & S_{-}\sin\vartheta_{2} & S_{-}\cos\vartheta_{2}\\ S_{-}\sin\vartheta_{2} & 0 & 0\\ S_{-}\cos\vartheta_{2} & 0 & 0 \end{array}\right).$$

Taking into account the remaining part of the Hamiltonian (39) will lead to incorrect results in the future. Transformation parameters $\vartheta_{1,2}$ depend on time for the nonstationary Hamiltonian, but we apply the “adiabatic representation” [21] modified for a three-level system, where all non-diagonal elements are equal to zero. Only in this case can the rate of varying parameters describe adiabatic transitions between states and energy levels. If we take into account non-diagonal matrix elements (39), we will obtain corrections to states and energy levels within the framework of *standard* perturbation theory. For an exact solution, there can be no transitions other than adiabatic ones. So, for these reasons, we cannot take into account the last nonzero matrix elements (39) and assume them to be zero, as with the exact solution.

The procedure can be applied independently of the rate of the field change and, thus, independently of the applicability of the adiabatic approximation; however, the time-dependent eigenstates and energy eigenvalues retain clear physical meanings, just as with the evolution of the initial ones, only when the adiabatic approximation is applicable. That is, if the system is prepared in an eigenstate of the Hamiltonian at the initial time, the system remains in the evolved eigenstate of the Hamiltonian at time *t* only if the variation of the magnetic field is sufficiently slow to satisfy the adiabatic approximation, i.e.,

(40) $$\frac{\dot{\omega }\left(t\right)}{\omega^{2}\left(t\right)}\ll 1,\;\mathrm{where}\;\dot{\omega }\left(t\right)=\frac{d}{dt}\omega \left(t\right).$$

Thus, the unitary transformation (38) is treated as a ‘quasi-interaction representation’, similar to the standard interaction representation, but uses the unitary operator of Equation (38) instead of the unperturbed evolution operator:

(41) $$|\chi \left(t\right)\rangle ={\hat{T}}_{t}\left(t\right)|\varphi \left(t\right)\rangle ,\;|\varphi \left(0\right)\rangle ={\hat{T}}_{t}^{†}\left(0\right)|\chi \left(0\right)\rangle ,$$

The second Equation (41) is necessary because ${\hat{T}}_{t}\left(0\right)\neq \hat{1}$. The insertion of this transformation into Equation (37) gives the following (note that, hereinafter, we omit argument *t* in the transformation matrix ${\hat{T}}_{t}\left(t\right)$):

$$i{\dot{\hat{T}}}_{t}|\varphi \left(t\right)\rangle +i{\hat{T}}_{t}|\frac{\partial }{\partial t}\varphi \left(t\right)\rangle =\hat{H}{\hat{T}}_{t}|\varphi \left(t\right)\rangle$$

and, multiplying both sides of this equation by ${\hat{T}}^{†}\left(t\right)$, we obtain

(42) $$i\frac{\partial }{\partial t}|\varphi \left(t\right)\rangle ={\hat{T}}_{t}^{†}\hat{H}{\hat{T}}_{t}|\varphi \left(t\right)\rangle -i{\hat{T}}_{t}^{†}{\dot{\hat{T}}}_{t}|\varphi \left(t\right)\rangle .$$

The derivative ${\dot{\hat{T}}}_{t}$ is represented by the sum of two terms ${\dot{\hat{T}}}_{t}={\dot{\hat{T}}}_{1}{\hat{T}}_{2}+{\hat{T}}_{1}{\dot{\hat{T}}}_{2}$ and we obtain

(43) $${\hat{T}}_{t}^{†}{\dot{\hat{T}}}_{t}={\hat{T}}_{2}^{†}{\hat{T}}_{1}^{†}{\dot{\hat{T}}}_{1}{\hat{T}}_{2}+{\hat{T}}_{2}^{†}{\dot{\hat{T}}}_{2},$$

where we take into account ${\hat{T}}_{1}^{†}{\hat{T}}_{1}=1$.

The obtained matrix has only non-diagonal elements and is equal to the following expression:

(44) $${\hat{T}}_{t}^{†}{\dot{\hat{T}}}_{t}=\left(\begin{array}{ccc} 0 & -\cos\vartheta_{2}{\dot{\vartheta }}_{1} & \sin\vartheta_{2}{\dot{\vartheta }}_{1}\\ \cos\vartheta_{2}{\dot{\vartheta }}_{1} & 0 & -{\dot{\vartheta }}_{2}\\ -\sin\vartheta_{2}{\dot{\vartheta }}_{1} & {\dot{\vartheta }}_{2} & 0 \end{array}\right).$$

The explicit form for derivatives ${\dot{\vartheta }}_{1}$ and ${\dot{\vartheta }}_{2}$ are given in Appendix A.

Now, we can rewrite Equation (42) in the following form

(45) $$i\frac{\partial }{\partial t}|\varphi \left(t\right)\rangle =\left({\tilde{\hat{H}}}_{0}\left(t\right)+\tilde{\hat{V}}\left(t\right)\right)|\varphi \left(t\right)\rangle ,$$

where ${\tilde{\hat{H}}}_{0}\left(t\right)$ is a diagonal matrix that is dependent on the time energy levels, and perturbation is equal to

(46) $$\tilde{\hat{V}}\left(t\right)=-i{\hat{T}}_{t}^{†}{\dot{\hat{T}}}_{t}=\left(\begin{array}{ccc} 0 & i\cos\vartheta_{2}{\dot{\vartheta }}_{1} & -i\sin\vartheta_{2}{\dot{\vartheta }}_{1}\\ -i\cos\vartheta_{2}{\dot{\vartheta }}_{1} & 0 & i{\dot{\vartheta }}_{2}\\ i\sin\vartheta_{2}{\dot{\vartheta }}_{1} & -i{\dot{\vartheta }}_{2} & 0 \end{array}\right).$$

## 7. Transitions between Pseudo-Qutrit Energy Levels and States

The transitions between energy levels or states of a pseudo-qutrit in varying magnetic fields could be easily calculated in the first order of the nonstationary perturbation theory. Note that the transition probabilities between $|\varepsilon_{2}\rangle$ and $|\varepsilon_{3}\rangle$ states are proportional to the rate of the external magnetic field change defined by ${\dot{\vartheta }}_{2}$ and are very slow in the adiabatic approach. The transition probabilities between $|\varepsilon_{1}\rangle$ and $|\varepsilon_{2,3}\rangle$ are defined as ${\dot{\vartheta }}_{1}$ and ${\dot{\vartheta }}_{2}$.

To solve the problem, in the following, we need to transfer the usual interaction representation in Equation (45). This transfer is simple because of a diagonal form of the unperturbed Hamiltonian ${\tilde{\hat{H}}}_{0}\left(t\right)$ and a diagonal form of the evolution operator, respectively. These nonzero matrix elements are equal to

(47) $$U_{ii}\left(t\right)=\exp\left\{-i\int_{0}^{t}\varepsilon_{i}\left(t^{\prime }\right)dt^{\prime }\right\},$$

where $\varepsilon_{i}\left(t^{\prime }\right)$ are diagonal elements of the Hamiltonian ${\tilde{\hat{H}}}_{0}\left(t\right)$, i.e., “quasi-levels” of the pseudo-qutrit.

The perturbation matrix $\tilde{\hat{V}}\left(t\right)$ only has non-diagonal matrix elements, and in the interaction representation, they are equal to

(48) $${\left\{{\tilde{\hat{V}}}_{I}\left(t\right)\right\}}_{ik}=U_{ii}^{*}\left(t\right){\tilde{\hat{V}}}_{ik}U_{kk}\left(t\right)={\tilde{\hat{V}}}_{ik}\left(t\right)\exp\left\{i\int_{0}^{t}\omega_{ik}\left(t^{\prime }\right)dt^{\prime }\right\},$$

where $\omega_{ik}\left(t^{\prime }\right)=\varepsilon_{i}\left(t^{\prime }\right)-\varepsilon_{k}\left(t^{\prime }\right)$ is a corresponding transition frequency.

Now, we can write transition probabilities between energy levels $|\varepsilon_{i}\left(t\right)\rangle \equiv |\varphi_{i}\left(t\right)\rangle$ of the pseudo-qutrit using a standard relation:

(49) $$W_{ik}\left(t\right)={\left|\int_{0}^{t}{\tilde{\hat{V}}}_{ik}\left(t^{\prime }\right)\exp\left\{i\int_{0}^{t^{\prime }}\omega_{ik}\left(t^{\prime \prime }\right)dt^{\prime \prime }\right\}dt^{\prime }\right|}^{2}.$$

To determine the time evolution of states, we need to use the evolution operator of Schrödinger Equation (37). Since two types of transformations are used, the evolution operator should be defined as follows

$$|\chi \left(t\right)\rangle ={\hat{T}}_{t}\left(t\right)|\varphi \left(t\right)\rangle ={\hat{T}}_{t}\left(t\right){\hat{U}}_{t}\left(t\right)|\varphi \left(0\right)\rangle .$$

Here, ${\hat{U}}_{t}\left(t\right)$ is the “unperturbed” evolution operator of Equation (45) and is represented by a diagonal Matrix (47). Taking into account that $|\varphi \left(0\right)\rangle ={\hat{T}}_{t}^{†}\left(0\right)|\chi \left(0\right)\rangle$, we obtain

(50) $$|\chi \left(t\right)\rangle ={\hat{U}}_{0}\left(t\right)|\chi \left(0\right)\rangle ,\;\mathrm{where}\;{\hat{U}}_{0}\left(t\right)={\hat{T}}_{t}\left(t\right){\hat{U}}_{t}\left(t\right){\hat{T}}_{t}^{†}\left(0\right).$$

The evolution of pseudo-qutrit states in two initial states can be prepared experimentally

$${|\chi \left(0\right)\rangle }_{1}=|+\rangle |+\rangle =|q_{1}{\rangle \;\mathrm{and}\;|\chi \left(0\right)\rangle }_{2}=|+\rangle |-\rangle =\frac{1}{\sqrt{2}}(|q_{3}\rangle +|0,0\rangle ).$$

Corresponding initial states of the transformed Hamiltonian can be written as superpositions (note that the singlet state |0,0〉 is not mixed with the pseudo-qutrit states):

(51) $$\begin{array}{cc} {|\varphi \left(0\right)\rangle }_{1} & =\cos\vartheta_{1}\left(0\right)|\varepsilon_{1}\rangle -\sin\vartheta_{1}\left(0\right)\cos\vartheta_{2}\left(0\right)|\varepsilon_{2}\rangle +\sin\vartheta_{1}\left(0\right)\sin\vartheta_{2}\left(0\right)|\varepsilon_{3}\rangle ,\\ {|\varphi \left(0\right)\rangle }_{2} & =\frac{1}{\sqrt{2}}(\sin\vartheta_{2}\left(0\right)|\varepsilon_{2}\rangle +\cos\vartheta_{2}\left(0\right)|\varepsilon_{3}\rangle ). \end{array}$$

In the following, we need to introduce some notations to make expressions for ${|\chi \left(t\right)\rangle }_{1,2}$ more compact and readable. Namely, we define four depending on the time coefficients:

(52) $$\begin{array}{cc} \cos\vartheta_{1,2}\left(t\right)\cos\vartheta_{1,2}\left(0\right)=a^{\left(1,2\right)}\left(t\right), & \;\sin\vartheta_{1,2}\left(t\right)\sin\vartheta_{1,2}\left(0\right)=b^{\left(1,2\right)}\left(t\right),\\ \cos\vartheta_{1,2}\left(t\right)\sin\vartheta_{1,2}\left(0\right)=c^{\left(1,2\right)}\left(t\right), & \;\sin\vartheta_{1,2}\left(t\right)\cos\vartheta_{1,2}\left(0\right)=d^{\left(1,2\right)}\left(t\right) \end{array}$$

and the corresponding four functions:

(53) $$\begin{array}{cc} a^{\left(2\right)}\left(t\right)e^{-i\int_{0}^{t}\varepsilon_{2}\left(t^{\prime }\right)dt^{\prime }}+b^{\left(2\right)}\left(t\right)e^{-i\int_{0}^{t}\varepsilon_{3}\left(t^{\prime }\right)dt^{\prime }}=f_{1}\left(t\right), & \;b^{\left(2\right)}\left(t\right)e^{-i\int_{0}^{t}\varepsilon_{2}\left(t^{\prime }\right)dt^{\prime }}+a^{\left(2\right)}\left(t\right)e^{-i\int_{0}^{t}\varepsilon_{3}\left(t^{\prime }\right)dt^{\prime }}=f_{2}\left(t\right),\\ c^{\left(2\right)}\left(t\right)e^{-i\int_{0}^{t}\varepsilon_{2}\left(t^{\prime }\right)dt^{\prime }}-d^{\left(2\right)}\left(t\right)e^{-i\int_{0}^{t}\varepsilon_{3}\left(t^{\prime }\right)dt^{\prime }}=f_{3}\left(t\right), & \;d^{\left(2\right)}\left(t\right)e^{-i\int_{0}^{t}\varepsilon_{2}\left(t^{\prime }\right)dt^{\prime }}-c^{\left(2\right)}\left(t\right)e^{-i\int_{0}^{t}\varepsilon_{3}\left(t^{\prime }\right)dt^{\prime }}=f_{4}\left(t\right). \end{array}$$

Using notations (52) and (53), we can write states ${|\chi \left(t\right)\rangle }_{1}$ and ${|\chi \left(t\right)\rangle }_{2}$ as follows:

(54) $$\begin{array}{cc} {|\chi \left(t\right)\rangle }_{1}= & \left(a^{\left(1\right)}\left(t\right)e^{-i\int_{0}^{t}\varepsilon_{1}\left(t^{\prime }\right)dt^{\prime }}+b^{\left(1\right)}\left(t\right)f_{1}\left(t\right)\right)|q_{1}\rangle +\left(d^{\left(1\right)}\left(t\right)e^{-i\int_{0}^{t}\varepsilon_{1}\left(t^{\prime }\right)dt^{\prime }}-c^{\left(1\right)}\left(t\right)f_{1}\left(t\right)\right)|q_{2}\rangle -\sin\vartheta_{1}\left(0\right)f_{4}\left(t\right)|q_{3}\rangle \\ {|\chi \left(t\right)\rangle }_{2}= & \frac{1}{\sqrt{2}}(-f_{3}\left(t\right)\left(\sin\vartheta_{1}\left(t\right)|q_{1}\rangle -\cos\vartheta_{1}\left(t\right)|q_{2}\rangle \right)+f_{2}\left(t\right)|q_{3}\rangle ). \end{array}$$

We can see that the separable initial state ${|\chi \left(0\right)\rangle }_{2}$ and ${|\chi \left(0\right)\rangle }_{1}$ represent complete superpositions of the base states of (14) at an arbitrary moment in time and cannot be represented as states with certain quasi-energy levels.

## 8. Discussion

Equations (49) and (54) give us the opportunity to examine both transitions between energy levels of the pseudo-qutrit and determine the probabilities of separable or entangled states of two spins in varying external magnetic fields.

For numerical calculations, we used a model of an adiabatically varying external magnetic field in the form of

(55) $$x\left(t\right)=x_{0}+\delta x\left(t\right),\;\delta x\left(t\right)=a\tanh\left(\alpha \tilde{t}\right),$$

where $x_{0}$ is the initial static field, the parameter α<1, and the dimensionless time $\tilde{t}=\Omega t$. In the following, we choose α=0.1. The parameter *a* determines the limit of changing the external field. Hereinafter, we take a=1. For an adiabatically varying magnetic field, one needs to choose α≪1, when the model coincides with the Landau–Majorana–Stuckelberg–Zener (LMSZ) model [27,28,29,30] widely used in various problems with a time-dependent Hamiltonian [12,31,32,33,34,35].

**Figure 2:**
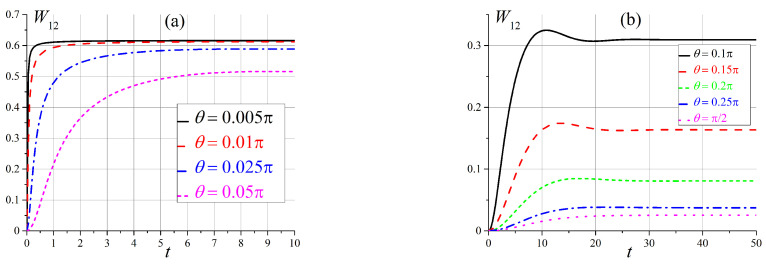
Time dependence of transition probabilities $W_{12}\left(t\right)$ (**a**) for small angles θ and (**b**) for θ>0.1π.

The most interesting situation for us is when the energy levels of the system are close to each other and we have a quasi-degenerate case. This picture is observed in the zero and intermediate (x≳1) magnetic fields. First, we consider transitions in a slowly varying magnetic field for $x_{0}=0$ between states of a doubly-degenerate energy level in the zero magnetic field, namely, between states $|\varepsilon_{1}\rangle$ and $|\varepsilon_{2}\rangle$, as seen in Figure 1. Note, that all transition probabilities are zero for the case when the magnetic field B∥n, i.e., θ=0. This situation is clear because all states are pure. The calculated transition probabilities $W_{12}\left(t\right)$ for some angles θ are shown in Figure 2a (hereinafter, angle θ is measured in radians). We see that transition probabilities show rapid growth, and they all tend to an asymptotic value equal to ≈0.6. This behavior is far from being exponentially slow due to the quasi-degeneration of energy levels. These results are in good agreement with the results of Section 2. In cases where the angle θ≈π/2, the transition probability $W_{12}\left(t\right)$ is small and tends to zero. The transition probability exhibits non-monotonic behavior for intermediate angles, but in all cases, it tends to a constant that depends on the angle θ, as shown in Figure 2b.

**Figure 3:**
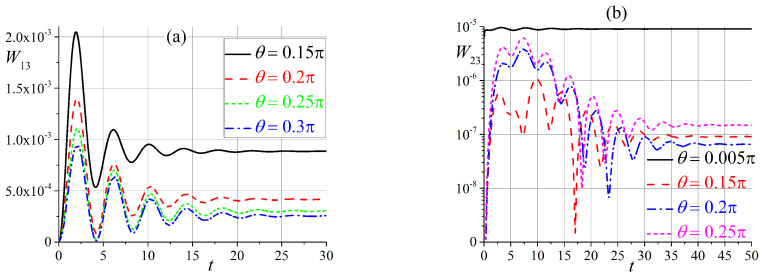
Time dependence of transition probabilities (**a**) $W_{13}\left(t\right)$, and (**b**) $W_{23}\left(t\right)$.

The transition probabilities $W_{13}\left(t\right)$ and $W_{23}\left(t\right)$ show clear oscillations, are exponentially small for all angles θ, and tend to angle-dependent constants, as shown in Figure 3. Note that both $W_{13}\left(t\right)$ and $W_{23}\left(t\right)$ are equal to zero for θ=0 and π/2.

**Figure 4:**
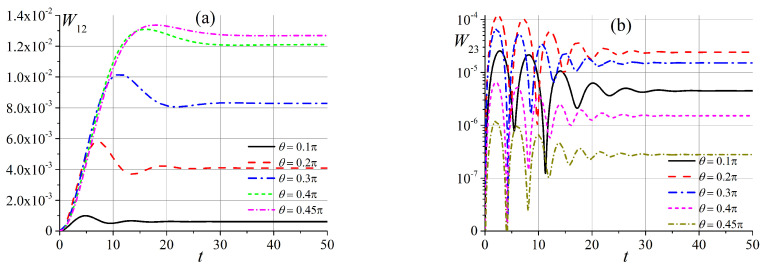
Time dependence of transition probabilities in an external magnetic field for $x_{0}=1$ (**a**) $W_{12}\left(t\right)$ and (**b**) $W_{23}\left(t\right)$.

As can be seen in Figure 1, in an intermediate magnetic field, when x∼6, energy levels $\varepsilon_{2}$ and $\varepsilon_{3}$ are close to each other, and the appropriate unperturbed states should be mixed. First, we compare the transition probabilities $W_{12}$ and $W_{23}$ in an external magnetic field at x∼1, the time dependence of which is shown in Figure 4. We can see that the transition probability $W_{12}$ is now small and increases when the angle θ tends to π/2. This is the opposite of a situation involving a small magnetic field (see Figure 2b).

**Figure 5:**
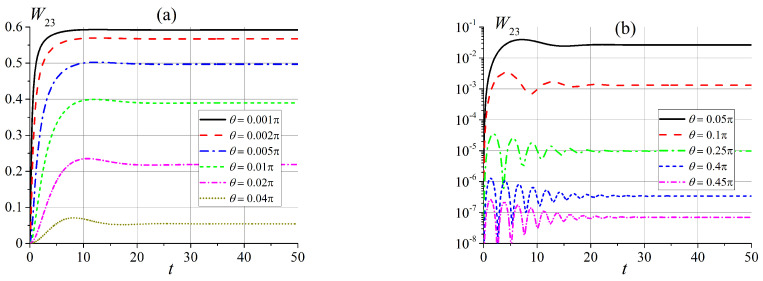
Time dependence of transition probabilities $W_{23}\left(t\right)$ in an external magnetic field for $x_{0}=6$ (**a**) for small angles θ, and (**b**) for angles θ>0.05π.

In a magnetic field, x≈6, the picture for the transition probability $W_{23}$ is similar to the picture for the transition probability $W_{12}$ in a weak magnetic field, as shown in Figure 5. Indeed, in this case, states $|\varepsilon_{2}\rangle$ and $|\varepsilon_{3}\rangle$ correspond to closely spaced energy levels. For an external magnetic field x>6, the transition probabilities of both $W_{12}$ and $W_{13}$ are exponentially small, they exhibit oscillations with decreasing amplitudes and tend to constants, depending on the angle θ.

**Figure 6:**
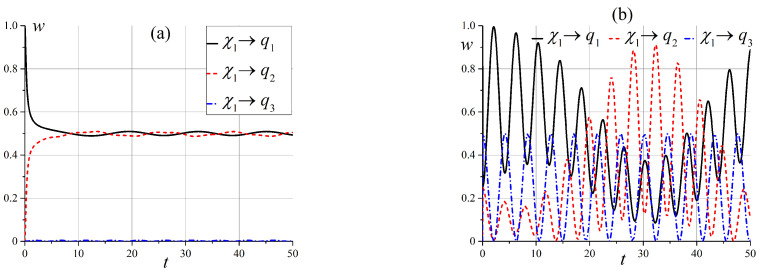
Time dependence of probabilities to observe separable states $w_{q_{i}}\left(t\right)$ when $x_{0}=0$ (**a**) for θ=0.025π, (**b**) and for θ=0.25π.

Now, let us analyze the behaviors of the state (54) by observing one of the separable states (14). It is easy to see that states ${|\chi \left(t\right)\rangle }_{1}$ and ${|\chi \left(t\right)\rangle }_{2}$ are entangled states at an arbitrary point in time, despite the fact that at the initial moment, the system was in a separable state. It would be interesting to know the moment when these states could be as separable as possible. In the following, we will consider the cases corresponding to those discussed above. At first, we illustrate the time-dependent probabilities $w_{q_{i}}$ to observe one of the separable states (14) at the zero initial magnetic field. Firstly, let us analyze state ${|\chi \left(t\right)\rangle }_{1}$. Note that for angle θ=0, all probabilities are zero, except for the probability of the initial state, which is 1. Figure 6 shows the behaviors of the probabilities over time for small and intermediate angles θ when the initial state is $|q_{1}\rangle$.

Small angle θ states $|q_{1}\rangle$ and $|q_{2}\rangle$ are completely entangled with small oscillations. For the intermediate angle, we have the maximum entanglement, but the oscillation amplitude is very large, and we can observe a situation with almost-separable states $|q_{1}\rangle$ and $|q_{2}\rangle$. At any given time, all three states are entangled.

**Figure 7:**
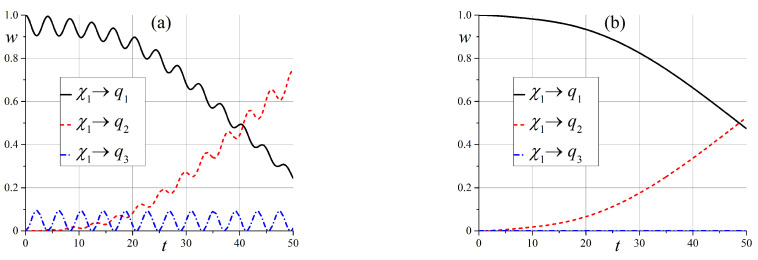
Time dependence of probabilities to observe separable states $w_{q_{i}}\left(t\right)$ in the case of the initial state $|q_{1}\rangle$ when $x_{0}=0$ (**a**) for θ=0.4π, and (**b**) for θ=π/2.

The pattern is very different for an angle close to π/2, as shown in Figure 7. For θ=π/2, we observe oscillations of only two entangled states $|q_{1}\rangle$ and $|q_{2}\rangle$ and transitions from maximally entangled to separable states.

**Figure 8:**
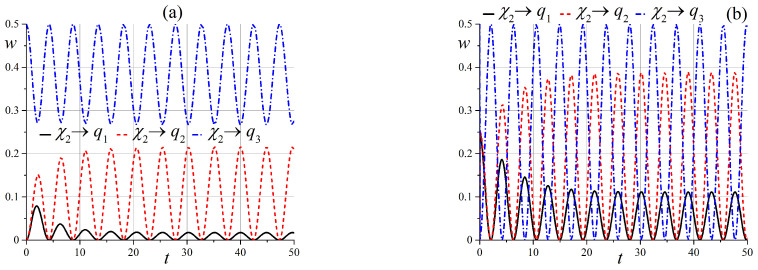
Time dependence of probabilities to observe separable states $w_{q_{i}}\left(t\right)$ in the case of the initial state $|q_{3}\rangle$ when $x_{0}=0$ (**a**) for θ=0.15π, and (**b**) for θ=π/4.

For state ${|\chi \left(t\right)\rangle }_{2}$, the pattern is simpler. For θ=0 and π/2, we have ${|\chi \left(t\right)\rangle }_{2}=f_{2}\left(t\right)|q_{3}\rangle$. If θ≠0,π/2 we observe periodical oscillations, and can obtain separable state $|q_{3}\rangle$, as shown in Figure 8.

Now, we will discuss the behavior of states in an intermediate static magnetic field when $x_{0}≳6$.

**Figure 9:**
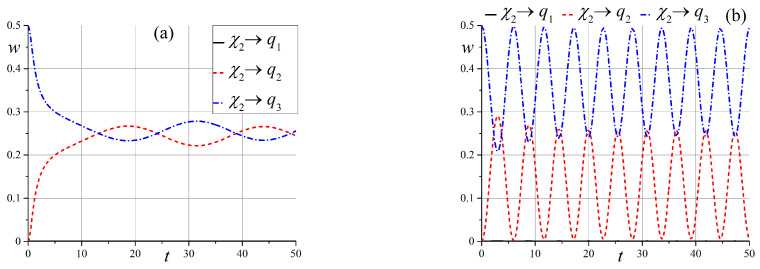
Time dependence of probabilities, which shows the entanglement of states $w_{q_{3}}\left(t\right)$ and $w_{q_{2}}\left(t\right)$ in the case of the initial state $|q_{3}\rangle$ when $x_{0}=6$ (**a**) for $\theta =1.5\cdot 10^{-2}\pi$, and (**b**) for θ=0.15π.

**Figure 10:**
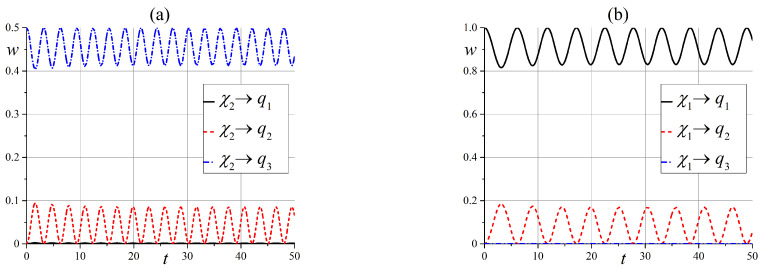
Time dependence of probabilities to observe the entanglement of states $w_{q_{i}}\left(t\right)$ when $x_{0}=6$ (**a**) in the case of the initial state $|q_{3}\rangle$ for θ=0.3π, and (**b**) in the case of the initial state $|q_{1}\rangle$ for θ=0.45π.

The behavior of state (54) in an intermediate external field is simpler compared to the case of a zero magnetic field. Indeed, the diagram of energy levels, which is given in Figure 1, shows that only two of the three states are effectively entangled. However, the entanglement depends on the angle θ: states $|q_{3}\rangle$ and $|q_{2}\rangle$ are entangled at small angles, and states $|q_{2}\rangle$ and $|q_{1}\rangle$ are entangled when θ→π/2. These results are illustrated in Figure 9 and Figure 10.

## 9. Conclusions

In this article, we examined the quantum dynamics of a pseudo-qutrit, which was formed in a system of two identical (but distinguishable) spins of 1/2, interconnected by a dipole–dipole interaction, and located in an external magnetic field that depends on time. We obtained an approximate analytical solution for the energy levels of the system, which was in good agreement with accurate numerical calculations. The obtained analytical results make it possible to develop the adiabatic representation introduced in [21] to describe the quantum dynamics of a pseudo-qutrit. The representation provides a very clear understanding of the time dependence for the transition rates between energy levels of a qutrit and the entanglement of two-qubit states. In particular, using the adiabatic representation, it is possible to describe transitions for a degenerate or quasi-degenerate energy spectrum.

The results can be applied to more complex systems with a time-dependent Hamiltonian, particularly for open systems, or for systems with non-Hermitian Hamiltonians. In the future, it will be useful to formulate the problem in terms of the density matrix formalism.

In this formalism, it is possible to use the probability representation of quantum states [16,20,36,37,38,39] and expressions of quantum states in terms of the Jordan–Schwinger map [40,41], where the spin states are given in the form of two-mode oscillator wave functions. This so-called Hermite polynomial representation of spin states [42,43,44] is formulated as the probability distribution of oscillator quantum states. The dynamics of the qutrit states discussed in our article can be formulated as dynamics of the probability distributions of two-mode oscillator states (both separable and entangled) using the integral of motion method of nonstationary oscillator states [45].

## Data Availability

All necessary data are contained in the article.

## Appendix A

Only the external magnetic field can vary in time, and derivatives ${\dot{\vartheta }}_{1}$ and ${\dot{\vartheta }}_{2}$ can depend on $\dot{x}$ only. Equation (28) gives the following relation:

$$\frac{d}{dt}\tan2\vartheta_{1}=\frac{2}{\cos^{2}2\vartheta_{1}}{\dot{\vartheta }}_{1}=-\frac{3y}{x^{2}}\dot{x}.$$

After simple transformations, we obtain a compact expression

(A1) $${\dot{\vartheta }}_{1}=-\frac{3y}{x^{2}+9y^{2}}\dot{x}.$$

The derivative for $\vartheta_{2}$ is relatively cumbersome, and we will give some main intermediate relations. Firstly, using definition (35), we can write

$$\frac{d}{dt}\tan2\vartheta_{2}=\frac{2\sqrt{2}\sin2\theta }{3\left(2-3y\right)-\sqrt{x^{2}+9y^{2}}}\left({\dot{S}}_{+}+\frac{S_{+}}{3\left(2-3y\right)-\sqrt{x^{2}+9y^{2}}}\frac{x\dot{x}}{\sqrt{x^{2}+9y^{2}}}\right).$$

The derivative of $S_{+}$ has a very compact view:

$${\dot{S}}_{+}=-\frac{1}{2}S_{-}\frac{3y}{x^{2}+9y^{2}}\dot{x}.$$

Now, we can write

$$\frac{d}{dt}\tan2\vartheta_{2}=2\left(1+\frac{8\sin^{2}2\theta S_{+}^{2}}{{\left(3\left(2-3y\right)-\sqrt{x^{2}+9y^{2}}\right)}^{2}}\right){\dot{\vartheta }}_{2}.$$

Note that for $S_{+}$, we have the following relation

$$S_{+}^{2}=1+\frac{3y}{\sqrt{x^{2}+9y^{2}}}.$$

Collecting the obtained relations, we have the final expression for the derivative of the second transformation parameter

(A2) $${\dot{\vartheta }}_{2}=\frac{2xS_{+}+3S_{-}y\left(1-\frac{3(2-y)}{\sqrt{x^{2}+9y^{2}}}\right)}{{\left(3\left(2-3y\right)-\sqrt{x^{2}+9y^{2}}\right)}^{2}\sqrt{x^{2}+9y^{2}}+8\left(\sqrt{x^{2}+9y^{2}}+3y\right)\sin^{2}2\theta }\cdot \frac{\sin2\theta }{2\sqrt{2}}\dot{x}.$$
